# Chiral 4-*O*-acylterpineol as transdermal permeation enhancers: insights of the enhancement mechanisms of a transdermal enantioselective delivery system for flurbiprofen

**DOI:** 10.1080/10717544.2020.1760403

**Published:** 2020-05-13

**Authors:** Tianzhe Chu, Chunyan Wang, Jing Wang, Heping Wang, Dandan Geng, Chensi Wu, Linlin Zhao, Ligang Zhao

**Affiliations:** a School of Pharmacy, North China University of Science and Technology, Tangshan, China;; b Department of Pharmacy, Tangshan Maternal and Child Health Hospital, Tangshan, China;; c Tangshan key laboratory of novel preparations and drug release technology, Tangshan, China

**Keywords:** Chiral 4-*O*-acylterpineol derivatives, permeation enhancers, transdermal drug delivery, the *in vitro* and *in vivo* correlation, penetration mechanism

## Abstract

In order to devise more effective penetration enhancers, 4-*O*-acylterpineol derivatives which were expected to be hydrolyzed into nontoxic metabolites by esterase in the living epidermis, were synthesized from 4-terpineol (4-TER) enantiomers and straight chain fatty acids. Their promoting activities on the *SR*-flurbiprofen and its enantiomers were tested across full-thickness rabbit skin, as well as to correlate under *in vitro* and *in vivo* conditions. The permeation studies indicated that both *d*-4-*O*-acylterpineol and *l*-4-*O*-acylterpineol had significant enhancing effects, interestingly, *d*-4-*O*-aclyterpineol had higher enhancing effects than *l*-4-*O*-aclyterpineol with the exception of *d*-4-methyl-1-(1-methylethyl)-3-cyclohexen-1-yl octadec-9-enoate (*d*-4-T-dC18). The mechanism of 4-*O*-acylterpineol facilitating the drug penetration across the skin was confirmed by Attenuated total reflection-Fourier transformed infrared spectroscopy (ATR-FTIR) and molecular simulation. The mechanism of penetration enhancers promoting drug release was explored by the *in vitro* release experiment. Finally, a relative safety skin irritation of enhancers was also investigated by *in vivo* histological evaluation. The present research suggested that *d*-4-*O*-aclyterpineol and *l*-4-*O*-aclyterpineol could significantly promote the penetration of *SR*-flurbiprofen and its enantiomers both *in vitro* and *in vivo*, with the superiorities of high flux and low dermal toxicity.

## Introduction

The transdermal drug delivery system (TDDS) had received much attention in the pharmaceutical industry during recent years with superiorities of extended therapeutic action, decreased side effects, convenient use and better patient compliance (Ashtikar et al., 2016). Stratum corneum (SC) which was composed of ceramides, cholesterol and free fatty acids is the main barrier against foreign bodies and the penetration of drugs (Chen et al., 2015b). In order to achieve reasonable bioavailability, the permeation enhancers were used to augment the permeability of drugs across the skin, which could avoid specific devices with high costs. To date, various permeation enhancers to enhance drug permeation across the skin have been investigated (Zhao et al., 2008, 2009; Chen et al., 2015a; Harada et al., 2018). Since ceramides (CER), the major lipid lamellae in SC, have two or more chiral carbons within the polar head, the SC formed essentially a chiral environment (Bouwstra et al., 1999; Masukawa et al., 2008; Wang and Klauda, 2019). Chiral compounds may have stereoselectivity under the influence of the chiral environment (Heard et al., 1993; Rong et al., 2014), therefore, the investigation of chiral compounds has been a critical issue in various domains including the TDDS.

Flurbiprofen (FP), which is still recommended to patients with arthritis and gout, is one of the non-steroidal anti-inflammatory analgesics drugs (Akhlaq et al., 2011). Transdermal preparation of FP can avoid the side effects of oral preparations, such as nausea, vomiting and peptic ulcers. It has been reported that enantioselectivity interaction was existed during transdermal permeation of FP across the rat skin (Valentová et al., 2010), the steady-state flux and permeability coefficient of *R*-FP were significantly higher than those of *S*-FP. However, the permeation difference between racemate and enantiomers of FP has not been reported so far.

Compared with other kinds of enhancers, 4-terpineol (4-TER), a natural cyclic terpene with a double bond, was more effective for delivering drugs through the skin. (Herman and Herman, 2015). However, the practical application of 4-TER in TDDS would be restricted by its volatile and undesirable odor. The aim of the present research was to develop new types of 4-*O*-acylterpineol derivatives to enhance transdermal delivery flurbiprofen (FP) racemate and its enantiomers more effectively through the skin.

The significance of chirality in transdermal delivery development has been reviewed (Reddy et al., 2000), and numerous of literatures have focused on differences in the transdermal permeability of racemate and its enantiomers, such as metoprolol (Kommuru et al., 1999) and propranolol (Suedee et al., 2008). However, few reports have been published to investigate the stereoselectivity of chiral enhancers and to explore the mechanism of the penetration of racemate and its enantiomers. Vávrová synthesized a pair of 6-aminohexanoic acid 2-octylester enantiomers with one chiral center as enhancers and confirmed no difference in the promoting ability of the 6-aminohexanoic acid 2-octyleste enantiomers. The above research that the enhancing potency of the chiral compound did not depend on their isomerism spatial arrangement (Vávrová et al., 2002). However, some interesting discrepancies between the above report and our investigation were observed. The present research is to investigate the different effects of *d*-4-*O*-aclyterpineol and *l*-4-*O*-aclyterpineol on transdermal delivery of *SR*-FP and its enantiomers both *in vitro* and *in vivo* to confirm its potential for stereoselectivity. Additionally, the *in vitro* and *in vivo* correlation (IVIVC) was calculated by deconvolution to verify the feasibility of the penetration enhancer *in vitro* penetration test, a good IVIVC is always desirable in the drug development and optimization of formulation (Kesisoglou et al., 2015).

The enhancement mechanisms of chemical permeation enhancers on percutaneous absorption process have been reported, such as acting on the lipids or proteins of skin to disturb the arrangement rationality and altering the solvent nature of skin to modify the transmittance of drugs into skin (Cohen-Avrahami et al., 2012; Wang and Meng, 2017). Simultaneously, there was a comparison on the permeability of chiral enhancers on the racemate and enantiomers of drug (Zhang et al., 2006; Guo et al., 2016). However, the different enhancement mechanisms of chiral penetration enhancers for drug penetration have not been confirmed. Therefore, it is essential to obtain an integrated comprehending of the mechanism for chiral enhancers in promoting process. In the present study, researchers determined the stereoselectivity of the penetration enhancer from the spatial structure level with the molecular simulation, and investigated the structural changes of polar and hydrophobic regions in lipid bilayers after skin was treated with different enhancer enantiomers with the ATR-FTIR to determine the potential mechanisms of these chiral enhancers. Finally, the skin irritation of enhancers was also evaluated.

This is the first time that systematic experiments have been carried out to demonstrate the effect and mechanism of chiral penetration enhancers on the penetration of racemate and enantiomers of drug, which is of importance for the application of chiral enhancers in clinical practice.

## Material and methods

### Chemicals and animals

DURO-TAK 87-235A, pressure sensitive adhesive (PSA), was obtained from Henkel Co., Ltd. (NJ, USA); isopropyl paraben was purchased from Beijing Xingjin Chemical Plant (Beijing, China); *SR*-FP was furnished by Wuhan Yuancheng Technology Development Co., Ltd. (Hubei, China); *S*-FP and *R*-FP were obtained from Toronto Research Chemicals (Canada); *d*-4-TER and *l*-4-TER were purchased from China National Pharmaceutical Group Co., Ltd. (Beijing, China); *n*-butyric acid, *n*-hexanoic acid, *n*-heptanoic acid, *n*-tetradeconic acid, oleic acid and dichloro sulfoxide were supplied by Jindong Tianzheng chemical reagent Co., Ltd. (Tianjin, China); methanol and acetonitrile of chromatographic grade were purchased from the fisher Co, Ltd. (NJ, USA); other chemical reagents were conformed to the standard grade.

### Synthesis of chirality terpinen-4-ol ester derivatives

The reaction sequences for the preparation of *d*-4-*O*-acylterpineol and *l*-4-*O*-acylterpineol are presented as follows (supporting information Figure S1): 0.046 mol fatty acid was dissolved in 0.098 mol dichloro sulfoxide, the reaction mixture was kept for 3 h at 60 °C, then added 0.032 mol 4-TER, and kept at 75 °C for 3 h. The product was extracted three times with ethyl acetate, and dried with anhydrous MgSO_4_, then the residue was purified by column chromatography on silica gel.

The structures of the 4-*O*-acylterpineol were confirmed by NMR (ARX-300, Bruker, Switzerland) and HPLC-MS (ZQ-2000, Waters, USA). The ^1^H NMR and MS data are as follows:

- *d*-4-methyl-1-(1-methylethyl)-3-cyclohexen-1-yl hexanoate (*d*-T-C6). ESI-MS *m/z*: 253.4 [M + H]^+^; ^1^H NMR(CDCl_3_), *δ*: 0.91 (t, 3H, 6′-H), 1.02–1.07 (m, 8H, 2′–5’CH_2_), 1.08–1.09 (d, each 3H, 9–10CH_3_), 1.2 6–1.36 (m, 1H, 8-H), 1.56 (s, 3H, 7-H), 1.60–1.61 (d, 2H, 6-H), 1.63–1.66 (t, 2H, 5-H), 3.56–3.60 (t, 2H, 2-H), 4.16 (dd, 1H, *J =* 11.4 Hz, 3-H). Yield: 79.5%.
- *l*-4-methyl-1-(1-methylethyl)-3-cyclohexen-1-yl hexanoate (*l*-T-C6). ESI-MS *m/z*: 253.4 [M + H]^+^; ^1^H NMR(CDCl_3_), *δ*: 0.91 (t, 3H, 6′-H), 1.03–1.06 (m, 8H, 2′–5’CH_2_), 1.08–1.10 (d, each 3H, 9–10CH_3_), 1.2 5–1.36 (m, 1H, 8-H), 1.54 (s, 3H, 7-H), 1.59–1.61 (d, 2H, 6-H), 1.63–1.65 (t, 2H, 5-H), 3.56–3.59 (t, 2H, 2-H), 4.14 (dd, 1H, *J =* 11.4 Hz, 3-H). Yield: 82.9%.
- *d*-4-methyl-1-(1-methylethyl)-3-cyclohexen-1-yl heptanoate (*d*-T-C7). ESI-MS *m/z*: 267.4 [M + H]^+^; ^1^H NMR(CDCl_3_), *δ*: 0.91 (t, 3H, 7′-H), 1.01–1.06 (m, 10H, 2′–6’CH_2_), 1.07–1.09 (d, each 3H, 9–10CH_3_), 1.2 4–1.36 (m, 1H, 8-H), 1.51 (s, 3H, 7-H), 1.58–1.61 (d, 2H, 6-H), 1.62–1.65 (t, 2H, 5-H), 3.57–3.60 (t, 2H, 2-H), 4.13 (dd, 1H, *J =* 11.4 Hz, 3-H). Yield: 81.3%.
- *l*-4-methyl-1-(1-methylethyl)-3-cyclohexen-1-yl heptanoate (*l*-T-C7). ESI-MS *m/z*: 267.4 [M + H]^+^; ^1^H NMR(CDCl_3_), *δ*: 0.91 (t, 3H, 7′-H), 1.01–1.04 (m, 10H, 2′–6’CH_2_), 1.07–1.10 (d, each 3H, 9–10CH_3_), 1.2 3–1.35 (m, 1H, 8-H), 1.54 (s, 3H, 7-H), 1.60–1.62 (d, 2H, 6-H), 1.63–1.65 (t, 2H, 5-H), 3.57–3.60 (t, 2H, 2-H), 4.15 (dd, 1H, *J =* 11.4 Hz, 3-H). Yield: 78.4%.
- *d*-4-methyl-1-(1-methylethyl)-3-cyclohexen-1-yl tetradecanoate (*d*-T-C14). ESI-MS *m/z*: 365.6 [M + H]^+^; ^1^H NMR(CDCl_3_), *δ*: 0.88 (t, 3H, 14′-H), 1.02–1.05 (m, 24H, 2′–13’CH_2_), 1.06–1.07 (d, each 3H, 9–10CH_3_), 1.2 2–1.27 (m, 1H, 8-H), 1.56 (s, 3H, 7-H), 1.58–1.59 (d, 2H, 6-H), 1.61–1.63 (t, 2H, 5-H), 3.55–3.59 (t, 2H, 2-H), 4.10 (dd, 1H, *J =* 14.4 Hz, 3-H). Yield: 84.6%.
- *l*-4-methyl-1-(1-methylethyl)-3-cyclohexen-1-yl tetradecanoate (*l*-T-C14). ESI-MS *m/z*: 365.6 [M + H]^+^; ^1^H NMR(CDCl_3_), *δ*: 0.90 (t, 3H, 14′-H), 1.02–1.04 (m, 24H, 2′–13’CH_2_), 1.05–1.07 (d, each 3H, 9–10CH_3_), 1.2 4–1.29 (m, 1H, 8-H), 1.54 (s, 3H, 7-H), 1.58–1.59 (d, 2H, 6-H), 1.60–1.63 (t, 2H, 5-H), 3.56–3.59 (t, 2H, 2-H), 4.13 (dd, 1H, *J =* 14.4 Hz, 3-H). Yield: 82.4%.
- *d*-4-methyl-1-(1-methylethyl)-3-cyclohexen-1-yl octadec-9-enoate (*d*-T-dC18). ESI-MS *m/z*: 421.7 [M + H]^+^; ^1^H NMR(CDCl_3_), *δ*: 0.91 (t, 3H, 18′-H), 1.02–1.06 (m, 22H, 3′–6′,11′–17’CH_2_), 1.07–1.09 (d, each 3H, 9–10CH_3_), 1.25–1.27 (m, 1H, 8-H), 1.58 (s, 3H, 7-H), 1.59–1.62 (d, 2H, 6-H), 1.63–1.65 (t, 2H, 5-H), 1.96–2.02 (t, 4H, 7′,10′-H), 2.25–2.27 (d, 2H, 2′-H), 3.57–3.60 (t, 2H, 2-H), 4.11 (dd, 1H, *J =* 14.4 Hz, 3-H), 5.48 (2H, t, *J =* 5.4 Hz, 8′–9′-H). Yield: 86.7%.
- *l*-4-methyl-1-(1-methylethyl)-3-cyclohexen-1-yl octadec-9-enoate (*l*-T-dC18). ESI-MS *m/z*: 421.7 [M + H]^+^; ^1^H NMR(CDCl_3_), *δ*: 0.89 (t, 3H, 18′-H), 1.02–1.06 (m, 22H, 3′–6′,11′–17’CH_2_), 1.07–1.10 (d, each 3H, 9–10CH_3_), 1.24–1.27 (m, 1H, 8-H), 1.58(s, 3H, 7-H), 1.59–1.61 (d, 2H, 6-H), 1.63––1.66 (t, 2H, 5-H), 1.96–2.02 (t, 4H, 7′,10′-H), 2.26–2.28 (d, 2H, 2′-H), 3.56–3.59 (t, 2H, 2-H), 4.13 (dd, 1H, *J =* 14.4 Hz, 3-H), 5.48 (2H, t, *J =* 5.4 Hz, 8′–9′-H). Yield: 85.2%.

### Determination of drug solubility

The excess *SR*-FP or its single enantiomer (0.01 g) were sealed to 10 mL of pure water at 25 °C in glass vials and waved for 48 h in constant temperature air bath oscillator until fully dissolved. Finally, the contents were passed through the 0.45 μm microporous membrane and centrifuged at 10,000 rpm for 5 min, then aliquots for the supernatant saturated solution were diluted and analyzed by HPLC. The experiments were performed in triplicate.

### Determination of drug oil-water partition coefficient (log P)

The same volume of water and *n*-octanol were mixed and shaken for 24 h at 25 °C, then the equal of water saturated *n*-octanol solution and *n*-octanol saturated aqueous solution through liquid-separation were obtained. The *SR*-FP or its single enantiomer (0.001 g) were added in the saturated solution, which were mixed to the ratio of 1:4, then shook for 24–48 h followed by stand for 24 h. (The shakings were in constant temperature air bath oscillator at 25 °C). Afterwards, the HPLC method was utilized to detect the concentration of the substance in the oil phase and the water phase.

### Preparation of rabbit skin

Male, Japanese big-eared rabbits weighting 2.5–3.0 kg were purchased from the animal experimental center of North China University of Science and Technology. The experimental process was in accordance with the ‘Ethical Guide for Laboratory Animal Investigation’ and was approved by the Animal Experimental Center Committee of North China University of Science and Technology. All efforts were made to minimize animal suffering and to limit the number of animals used.

The hair of back skin was removed by clipper and electrical shaver carefully when the rabbits were anesthetized with ether. Full thickness of back skin was excised after the rabbit was sacrificed by injecting air into the ear veins, and the subcutaneous fat tissue was carefully removed using the surgical scissor and scalpel. The integrity of the skin was confirmed by microscopic observation method. The treated rabbit skin was cut into a size of 1.5 cm × 1.5 cm and stored at –80 °C until use. Before use, it needed to be immersed in physiological saline for 20 min to thaw.

### Preparation of drug-in-adhesive patch

Drug-in-adhesive patch (drug loading of 5% by adhesive) was prepared by using solvent evaporation method. An appropriate amount of FP was dissolved in a suitable amount of ethyl acetate, which was later added to the adhesive and mixed thoroughly with a mechanical stirrer. The resulting solution was coated onto release liner (Scotch Pak^®^ 9744, 3M, USA) by using lab coating unit (6025, BLD Instruments Co. Ltd., Guangdong, China). The coated laminate was dried at 50 °C for 15 min. After the solvent had been removed, it was covered with backing laminate (Scotch Pak^®^ 9732, 3M).

### *In vitro* permeation experiment

Modified two-chamber diffusion cells with an effective diffusion area of 0.95 cm^2^ were employed in the *in vitro* permeation experiment. The cells were kept at 32 °C by the circulated water bath and stirred with magnetic bars at 400 rpm. The dermis side of the skin was attached to one-half of the side-by-side diffusion cells, the FP patch was pressed on the skin with the adhesive side facing the SC, and pH 7.4 PBS was used as the receptor medium. At predetermined time points (2, 4, 6, 8, 10, 12 and 24 h), the receptor solution of 2.0 mL was withdrawn and equal volume PBS was added to maintain a constant volume of receptor solution.

### Data analysis

The cumulative amount of each drug permeating per unit area (*Q*) versus time was plotted.

$$Q_{24h}=\left(3.5C_{i}+2.0\sum_{i=1}^{n-1}C_{i-1}\right)/A$$

*Q* was the cumulative skin permeation amount. *Ci* was the concentration in the receiver compartment at time *i*, *A* was the effective diffusion area of diffusion.

The steady-state flux (*J_s_*, µg/cm^2^/h) was calculated from the slope of linear region of the plot. The lag-time was determined by extrapolating the linear portion of the curve to the abscissa.

$$J_{s}={dQ}_{r}/{Ad}_{t}$$

Enhancement ratio (ER) was used to evaluate the activity of the enhancers, which is the ratio of *Q_24h_* with enhancer to *Q_24h_* without enhancer.

$$ER=Q_{24h(\text{with enhancer})}/Q_{24h(\text{without enhancer})}$$

### *In vivo* pharmacokinetic study

#### Intravenous administration

A certain amount of FP was dissolved in a mixed solution of sterile water and Tween-80 at 2.0 mg/mL. The fresh intravenous solution was prepared just before intravenous administration.

Six male rabbits weighing 2.5 kg were selected for IV. A capacity of 4.8 mg/kg FP (0.2 g FP was dissolved in a mixed solution of pure water and Tween80) was administered to the one ear vein of rabbit, the other ear vein of rabbit was phlebotomized about 0.5 mL at 5, 10, 15, 30 min and 1, 2, 3, 4, 6, 8, 10, 12 h. The blood samples were centrifuged at 4000 rpm for 10 min and kept at –80 °C.

#### Transdermal patch administration

Thirty-six male rabbits weighing 2.5 kg were divided into six groups, and the back skin was intact without erythema. For patch administration studies, patches without or with enhancers (120 mg/kg) were stuck to the shaved area of the rabbits. They were administered as follows: (1) control group: only the *SR*-FP; (2) enhancer group A: prepared *SR*-FP patch with 5% *d*-4-TER; (3) enhancer group B: prepared *SR*-FP patch with 5% *d*-4-T-dC18; (4) enhancer group C: prepared *SR*-FP patch with 5% *l*-4-T-dC18; (5) enhancer group D: prepared *S*-FP patch with 5% *d*-4-T-dC18; (6) enhancer group E: prepared *R*-FP patch with 5% *d*-4-T-dC18. In order to prevent the patches fallen off, gauze and medical tape were used to fix the patches. The blood from ear vein of rabbit was collected 0.5 mL at 0.5, 1, 2, 4, 6, 8, 12, 16, 20, 24, 26, 28, 30, 32, 36 and 48 h, and the patch was removed after 24 h, then the residual drug was wiped with physiological saline. The sample was processed by the same method as IV. All animal studies were carried out in accordance with the NIH Guidelines for the Care and Use of Laboratory Animals and were also approved by the Life Science Research Center of North China University of Science and Technology.

#### Preparation of samples

After being thawed and equilibrated to room temperature, an aliquot (100 μL) of plasma sample was spiked with isopropyl paraben (10 μL) of 40.0 μg/mL and 400 μL methanol. The samples were vortexed for 5 min and followed by centrifugation at 16,000 rpm for 10 min at 25 °C. After centrifugation, the supernatant was transferred to a clean tube and evaporated to dryness at 40 °C under a gentle stream of nitrogen. The dried samples were reconstituted with 100 μL mobile phase, vortexed and centrifuged before analysis.

#### Pharmacokinetic analysis

Pharmacokinetic parameters were calculated using WinNonlin^®^ software (Pharsight Corporation, San Diego, CA, USA) based on noncompartmental model, including the maximal plasma drug concentration (*C*_max_), the time to reach *C*_max_ (*T*_max_) which were obtained from the observed data, the area under the time–concentration curve from time 0 to time *t* (AUC_0–_*_t_*) of distribution which was calculated using the linear trapezoidal rule, the mean residence time (MRT), the plasma clearance (Cl) and the apparent volume(*V*). Enhancement factor (*E_f_*) was calculated from the following equation:

$$E_{f}={\mathrm{AUC}}_{0-t(\text{with enhancer})}/{\mathrm{AUC}}_{0-t(\text{without enhancer})}$$

##### IVIVC

For IVIVC studies, the Wagner-Nelson method was often used for single-chamber model calculations, the IAO-Riegelman method was often used for two-compartment model calculations, and the Deconvolution method was used for non-compartment models.

The absorption concentration of the drug *in vivo* could be expressed by the following volume integral equation:

$$C_{\left(t\right)}=\int_{0}^{t}R_{\left(\theta \right)}\cdot W_{\left(t-\theta \right)}d_{\theta }$$

*C* represented the output function, i.e., the concentration function of the patch administration; *W* represented the weight function, i.e., the concentration function for IV, expressed as the AUC of IV during this time interval; *R* represented the input function, i.e., the average absorption rate during this time interval.

The formula can be interpreted as:

$$C_{\left(ti\right)}=\sum_{k=1}^{k=i}R_{k}\cdot {\mathrm{AUC}}_{T_{i}-T_{k}}^{T_{i}-T_{k-1}}$$

So, for each measurement point *R*:

$$R_{1}=C_{1}/{\mathrm{AUC}}^{t_{1}}$$

$$R_{2}=\left(C_{2}-R_{1}\cdot {\mathrm{AUC}}_{t_{2}-t_{1}}^{t_{2}}\right)/{\mathrm{AUC}}^{t_{2}-t_{1}}$$

$$R_{3}=\left(C_{3}-R_{1}\cdot {\mathrm{AUC}}_{t_{3}-t_{1}}^{t_{3}}-R_{2}\cdot {\mathrm{AUC}}_{t_{3}-t_{2}}^{t_{3-}t_{1}}\right)/{\mathrm{AUC}}^{t_{3}-t_{2}}$$

……

The data was calculated in Microsoft Excel and finally cumulative permeability *in vivo* (*vivo*%) was obtained. The *in vitro* cumulative percentage (*vitro*%) was used as the independent variable, and *vivo*% was used as the dependent variable, correlation analysis was performed in GraphPad Prism 7.0.

#### Drug release experiments

In the drug release study, the drug dissolution apparatus (Xin Tianguang, Tianjin, China) was set at 32 ± 0.5 °C, whose effective contact area with the patch was 16 cm^2^. The patch was sandwiched in the double-layered round mesh disk and dipped into a dissolution cup with receptor fluid (phosphate buffer solution, pH7.4) of 900 mL (*n* = 6). Samples were withdrawn (5.0 mL) at the scheduled time point of 0.5, 1, 2, 4, 6, 8, 10, 12 and 24 h, and the same volume of phosphate buffer solution was replenished.

#### HPLC analysis of FP

The concentrations of drugs in the receptor medium or rabbit plasma were determined using a validated HPLC method. The HPLC system was equipped with a Shimadzu HPLC apparatus, including an LC-20AT pump, a SIL20A syringe, an SPD-20A detector, and a CTO-20A column oven. The propylparaben was used as an internal standard, and the flow rate of chromatographic analysis was set 1.0 mL/min. For *in vitro* analysis of *SR*-FP, thermo (USA) C18 column (150 mm × 4.6 mm, 5 μm particle) maintained at 40 °C was used to elute *SR*-FP, the mobile phase was used methanol and 1% aqueous glacial acetic acid (60:40, v/v) and the wavelength was set at 247 nm. The HPLC conditions of *S*-FP and *R*-FP were as follows: The column was CHIRALPAK®IA (4.6 mm × 250 mml, 5 μm particle diameter) of DAOCEL (Shanghai, China). The mobile phase consisted of acetonitrile and trifluoroacetic acid (0.05%, 50:50, v/v), the wavelength was set at 254 nm. For *in vivo* analysis of *S*-FP and *R*-FP, the mobile phase consisted of acetonitrile and trifluoroacetic acid (0.05%, 35:65, v/v), and other conditions were the same to *in vitro* analysis.

### Molecular simulation

Molecular simulation was used to predict the intermolecular interaction among the stratum corneum ceramide and enhancers. The structures of molecular were constructed by ChemDraw 16.0. By calculation with COMPASSII force field, the lowest energy conformations of compound and mixture were found in Materials Studio software 7.0 with Forcite and Blend. The mixing frequency was set at 1000, and all the process were calculated in Fine. Then docking calculations were performed with the Dreding force field, the corresponding energy was calculated (Wang et al., 2017).

#### 

##### ATR-FTIR

The shaved back was divided into 21 areas (each area in 1 cm^2^) and randomly divided into seven groups (*n* = 3). The marked areas were applied with 5% (w/v) enhancer solution of 20 μL. They were administered as follows: (1) control group only the absolute ethanol; (2) group A with *d*-4-TER; (3) group B with *l*-4-TER; (4) group C with *d*-4-T-C14; (5) group D with *l*-4-T-C14; (6) group E with *d*-4-T- dC18; and (7) group F with *l*-4-T-dC18. After the enhancer was infiltrated of 30 min, the skin of the penetrated area was peeled off. Then the residual solution was blotted up and dried of 15 min. Finally, the treated skin was placed on the surface of zinc selenide (ZnSe) with an incidence angle of 45° and detected by ATR-FTIR spectroscopy (Thermo Nicolet NEXUS 470, Madison, USA). The stratum corneum was scanned 200 times in the detection wavelength range of 1000–4000 cm^−1^ at a resolution of 2 cm^−1^. Infrared absorption peaks were analyzed using Oringe 7.0.

### Histological study

The back hair of rabbits was removed with the same method above, then the shaved back was divided into five areas (each area of 1 cm^2^). The marked areas were applied with 5% (w/v) enhancer solution of 20 μL. They were administered as follows: (1) control group (without enhancer); (2) group A with *d*-4-TER; (3) group B with *l*-4-TER; (4) group C with *d*-4-T-dC18 and (5) group D with *l*-4-T-dC18. After the enhancer was infiltrated for 1 h, the surface-relieving penetration enhancer was removed by alcohol cotton. Rabbits were sacrificed by injecting air into the ear veins, then the shaved skin was cut and fixed by 4% paraformaldehyde for one day. The skin was embedded in paraffin for 30 min under vacuum at 60 °C, subjected to paraffin vertical coronal sections and stained with HE. These samples were then observed under Olympus microscope and compared with the control sample that was free of exposure to drugs.

#### Statistical analysis

Statistical analysis was carried out using analysis of variance (ANOVA). Data was considered significant when *p* < .05. Correlation coefficients were examined for significance (*p* < .05) using Student’s *t*-test of the SPSS program.

## Results and discussion

### 

#### *In vitro* permeation study

When cumulative amounts permeating as a function of the enhancer concentration were compared at 24 h, the fluctuation was apparent. At 0%, 3% and 5% *d*-4-TER level (w/w), the cumulative transports of *SR*-FP were 122.93 ± 8.02 μg/cm^2^, 168.14 ± 19.95 μg/cm^2^ and 266.01 ± 58.81 μg/cm^2^, respectively. The cumulative transports of *SR*-FP were 145.16 ± 13.64 μg/cm^2^ and 222.09 ± 19.19 μg/cm^2^ for 3% and 5% *l*-4-TER level, respectively. At 7% level of chiral 4-TER, the enhancer was precipitated from the patch for its high concentration. It can be seen that at 5% concentration, the cumulative permeation of *SR*-FP was higher than the other levels (*p* < .05), so the 5% level was chosen to perform the latter experiment. The concentration of 4-*O*-acylterpineol derivatives during the subsequent experiments were at the same molar concentration with 4-TER which was selected for *SR*-FP.

**Figure 1.**
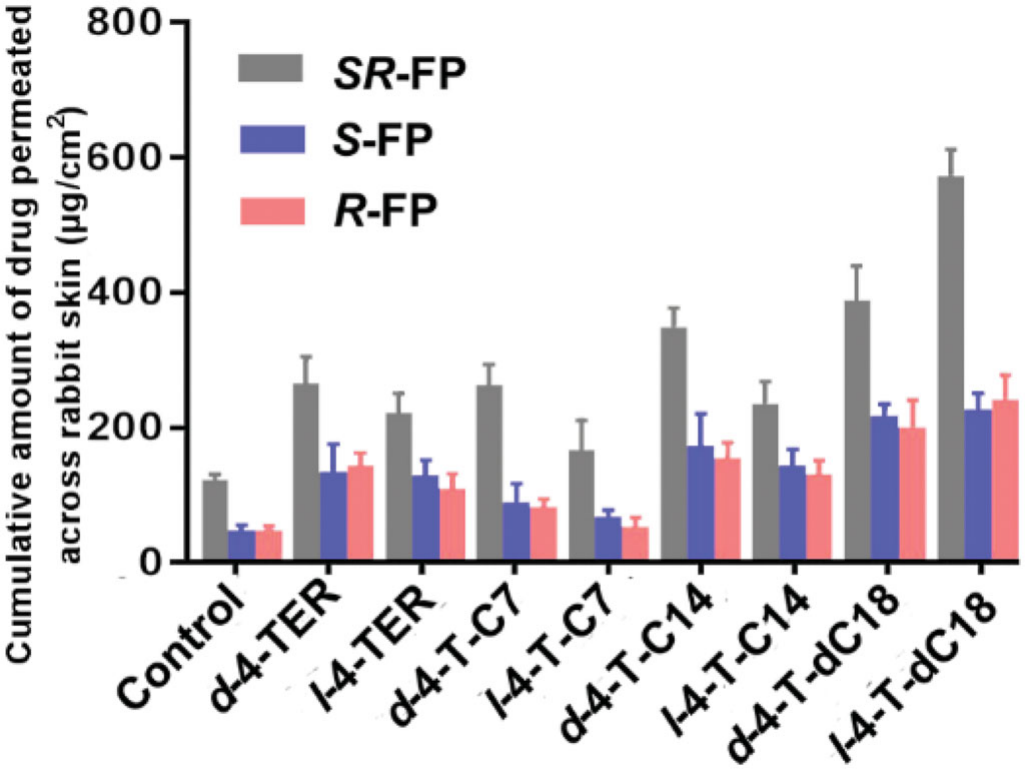
Permeation profiles of FP in patches with or without enhancers through rabbit skin (*n* = 4, mean ± SD), the amount of 4-*O*-acylterpineol is equal to the molar concentration of 4-TER.

The permeation parameters of *SR*-FP and its enantiomers (*J*_s_, *T*_lag_, ER and *Q*_24h_) with or without chiral 4-*O*-acylterpineol were listed in Table 1 and Figure 1. The *in vitro* experiment indicated that all the enhancers had significantly higher enhancing effects on the permeation of *SR*-FP and its enantiomers than the control group (*p* < .05). By comparison, *l*-4-T-dC18 had the most significant enhancing effect, and the attained *Q*_24h_ was 547.20 ± 35.94 μg/cm^2^, increasing the SR-FP in *Q*_24h_ by 4.45-fold. It was also found that esterification resulted in greater lipophilicity due to the masking of the hydroxyl group of chiral 4-TER, so the effect of ester promoting penetration is stronger than that of the equimolar amount of alcohol (Miyake et al., 2010). It may cause different penetration effects due to the length or structure of the tail carbon chain (Nasompag et al., 2015).

**Table 1.** Skin permeation parameters of the *SR*-FP and its enantiomers through excised rabbit skin from the patches containing 5% drugs (w/w).

Enhancer	*SR*-FP			*R*-FP			*S*-FP		
	*J_s_* (μg/cm^2^/h)	*T*_lag_(h)	ER	*J_s_* (μg/cm^2^/h)	*T*_lag_(h)	ER	*J_s_* (μg/cm^2^/h)	*T*_lag_(h)	ER
Control	5.54 ± 0.37	3.31	1.00	2.43 ± 0.18	2.43	1.00	2.65 ± 0.29	3.82	1.00
*d*-4-TER	14.43 ± 3.62*	3.60	2.16	7.34 ± 2.51*	3.58	2.74	7.60 ± 1.47*	2.45	2.97
*l*-4-TER	11.43 ± 2.06*	3.13	1.81	6.99 ± 1.12*	3.82	2.64	6.02 ± 0.99*	4.07	2.25
*d*-4-T-C7	14.46 ± 1.52*	3.74	2.14	4.40 ± 1.42*	2.40	1.96	4.39 ± 0.49*	2.91	1.69
*l*-4-T-C7	6.58 ± 1.41*	2.96	1.35	3.19 ± 0.47*	2.11	1.38	3.00 ± 0.69	4.18	1.10
*d*-4-T-C14	18.79 ± 3.81**	3.58	2.85	8.16 ± 0.84**	3.98	3.19	7.75 ± 1.68**	2.04	3.53
*l*-4-T-C14	11.83 ± 6.27**	2.45	1.92	7.62 ± 1.49**	3.51	2.92	6.80 ± 0.50**	3.37	2.73
*d*-4-T-dC18	17.57 ± 4.13**	1.85	2.77	11.81 ± 1.23**	3.95	4.43	10.99 ± 2.17**	4.08	4.13
*l*-4-T- dC18	20.53 ± 3.11**	3.61	4.45	12.04 ± 2.51**	3.68	4.6	12.98 ± 3.51**	3.89	4.98

Data are given as mean ± SD (*n* = 4).

Enhancement ratio (ER) calculated as follows: *Q*_24h_ (with enhancer)/*Q*_24h_ (without enhancer).

*Significantly different from control group, *p* < .05; **significantly different from terpineol group, *p* < .05.

Since *d*-4-*O*-acylterpineol and *l*-4-*O*-acylterpineol produced high skin permeation rates of *SR*-FP, their enhancement in the transdermal process of *S*-FP and *R*-FP were also investigated, and the results are shown in Figure 1 and Table 1. *d-*4-*O*-acylterpineol was more effective in elevating the percutaneous penetration of *SR*-FP than *l*-4-*O*-acylterpineol with the exception of 4-T-dC18, and similar significant differences in the effects of *d*-, *l*-4-TER were also observed on *Q*_24h_. The previous research has shown that the enhancing properties of the compounds are not dependent on their spatial arrangement, and the stereoselective interactions between an enhancer and stratum corneum components seem not to be important for the enhancer action (Vávrová et al. 2002). However, the effect of the spatial arrangement of enhancers on the penetration enhancement was confirmed by the present study, which displayed the significant differences in the effects of *d*-, *l*-4-TER. Consequently, the further investigation of chiral 4-*O*-acylterpineol at a molecular level should be proposed in order to elucidate the penetration mechanism more clearly.

Interestingly, the different promoting ability of *d*-4-T-dC18 and *l*-4-T-dC18 in comparison with the other *d*- and *l*-acylterpineol was observed in spite of the fact that they have the same polar head. It was reported that the different capabilities of chiral enhancers in the drug penetration can be elucidated by a different spatial configuration which is caused by the quantity and location of the double bonds (Clouet et al., 1989). Therefore, the different promoting ability of *d*-4-T-dC18 and *l*-4-T-dC18 could be assumed that the double bond in the oleic acid molecule could affect the spatial configuration of the molecule or change the physicochemical properties of the 4-*O*-acylterpineol.

In the present investigation, all enhancers had higher permeation amount of *SR*-FP and its enantiomers compared to the control group, however, their promoting abilities on *S*-FP and *R*-FP were almost identical (*p* > .05). Miyazaki et al. has reported different permeation rates of propranolol enantiomers through rat skin (Miyazaki et al., 1992). However, some other researchers reported that no enantiomeric differences existed in the permeation of propranolol through human skin (Heard et al. 1993). Valentova et al. has discussed the enantioselectivity of FP through rat skin, however, no FP enantiomers selectivity had been found through rabbit skin in the same report (Valentová et al., 2010). Therefore, it is assumed that the discrepancy in the previous literatures could be attributed to the different kinds and amounts of CERs which contained in the skin of different species. Additionally, when the same enhancer was employed, the penetration amounts of both *S*-FP and *R*-FP were significantly lower than those of *SR*-FP (*p* < .05). The solubilities of *SR*-FP, *R*-FP and *S*-FP were 89.11 ± 5.48, 49.82 ± 1.31 and 46.39 ± 4.30 μg/mL, respectively; The log*P* values of *SR*-FP, *R*-FP and *S*-FP were 2.28 ± 0.04, 1.83 ± 0.09 and 1.86 ± 0.05, respectively. The larger log*P* and solubility value of *SR*-FP may imply higher speed of permeant penetration across the SC (lipophilic layer) and the VE (hydrophilic layer) (Yamaguchi et al., 2008), which is essential to explain the transdermal process of FP. Previous literature also has shown that suitable solubility and log*P* value play an important role in transdermal absorption of drugs (Mrózek et al., 2011). The difference in physical properties between enantiomers and the racemic compound may also influence physiological phenomena, such as skin permeability, pharmacokinetics, and therapeutic effect (Ghosh and Reddy 2001). The above result suggested that the different permeation properties of the FP were not dependent on the spatial arrangement, instead of dependent on the difference in physical properties between enantiomers and the racemic compounds.

#### *In vivo* permeation study

Pharmacokinetic parameters after IV of FP in rabbits were required in order to establish the IVIVC correlation in the rabbits from the *in vitro* skin permeability data. However, there are no pharmacokinetic data available for FP in rabbits in the literature. Hence, pharmacokinetic parameters were calculated after intravenous administration of FP in rabbits.

**Figure 2.**
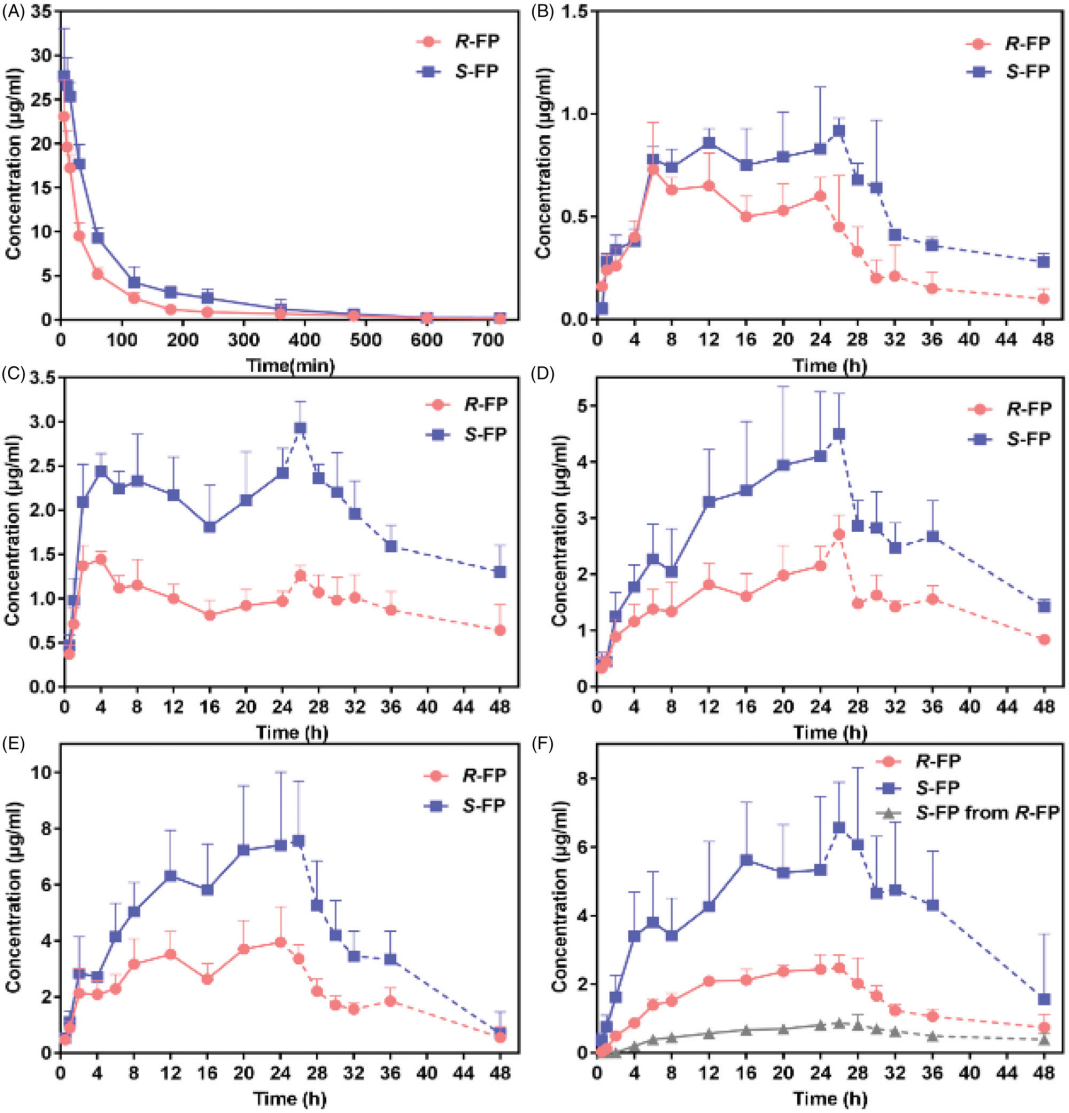
(A) Plasma concentration-time profiles of FP after intravenous injection in the rabbits; (B)–(F) Plasma concentration-time profiles in rabbits after transdermal application patches loaded FP racemate or enantiomer with or without enhancers; (B) control loaded FP racemate; (C) *d*-4-TER loaded FP racemate; (D) *d*-4-T-dC18 loaded FP racemate; (E) *l*-4-T- dC18 loaded FP racemate; (F) *d*-4-TER loaded enantiomers of FP (the dotted line represents the blood concentration after removing the patch) (*n* = 6, mean ± SE).

*R*-FP and *S*-FP were quickly absorbed and reached *C*_max_ at 1.71 ± 0.55 h and 1.66 ± 0.9 h after IV of 4.8 mg/kg *SR*-FP. The MRT values of *R*-FP and *S*-FP were 1.84 ± 0.15 h and 2.04 ± 0.23 h, the Cl values of *R*-FP and *S*-FP were 0.52 ± 0.03 L/h and 0.30 ± 0.02 L/h, respectively. This indicated that FP had a short retention time *in vivo* and eliminated quickly after IV. The pharmacokinetic study was successfully applied to the transdermal patches of *SR*-FP and its enantiomers in rabbits with or without chiral 4-*O*-acylterpineol. The plasma concentration-time profiles after the patch and IV were shown in Figure 2.

**Figure 3.**
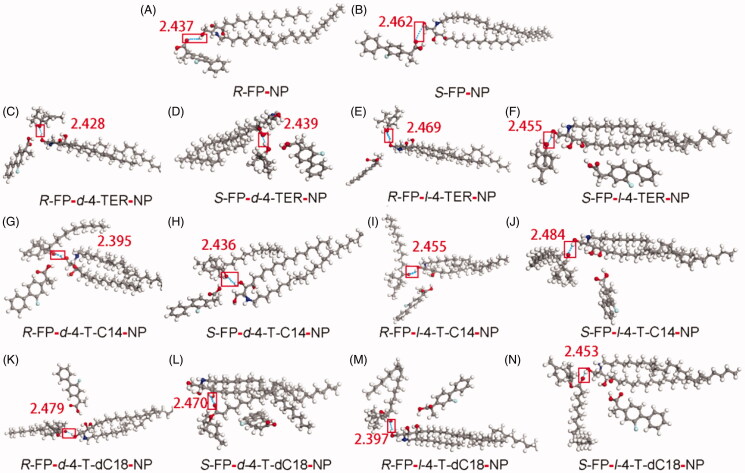
Interaction of the CER-NP assemblies with molecular models of drugs and enhancers. Carbon atoms were colored gray, oxygen atoms red, nitrogen atoms blue, hydrogen atoms white, fluorine atoms cyan. These figures are screenshots of the CER-NP assemblies. H-bonds were presented in light blue dotted lines.

In Figure 2(A), the plasma drug content of IV only continued about 10 h. Although patches were removed at 24 h, a few groups reached *C*_max_ after more than 24 h. Drugs could be maintained about 48 h in plasma which might be attributed to the reservoir effect of the skin. Additionally, a significant difference in the plasma concentration between the enantiomers after applying the *SR*-FP patch was observed (Figure 2(B–E)) (*p* < .05), which could be attributed to two possible reasons to explain this phenomenon. First, the elimination rate of *S*-FP was slower than that of *R*-FP (*p* < .05), which was available in the pharmacokinetic results of IV *in vivo*. This also has been confirmed in Figure 2(A). Additionally, the *S*-FP could be detected after administration of individual *R*-FP to rabbits, but inversion from *S*-FP to *R*-FP had not been observed, just as shown in Figure 2(F). However, there was almost no difference in the skin permeation of *S*-FP and *R*-FP during *in vitro* permeation experiments. These results demonstrated that the penetration process of the SC could not provide any enantiomer selectivity, which also has been illustrated by molecular simulation results, presented in Figure 3. Furthermore, as was shown might be attributed to the competitive elimination of enantiomers under the action of metabolizing enzyme in the body as previous reports (Lin et al., 2016).

The pharmacokinetic parameters were calculated in Table 2. The results of *in vivo* skin penetration study are similar to that of *in vitro*. *d*-4-T-dC18 and *l*-4-T-dC18 had significantly greater *C*_max_ and AUC_0-_*_t_* of FP than the control group (*p* < .05). Notably, the promoting ability of *l*-4-TER-dC18 was highest among all enhancers (*p* < .05). *l*-4-T-dC18 provided the highest AUC_0-_*_t_* and the maximum plasma concentration of *R*-FP (110.62 ± 21.62 h·μg/mL and 4.43 ± 1.03 μg/mL). Identically, *l-*4-T-dC18 provided the highest AUC_0-_*_t_* and the maximum plasma concentration of *S*-FP (209.04 ± 54.76 h·μg/mL and 8.27 ± 2.49 μg/mL). Despite the *in vitro* and *in vivo* permeability parameters were different attributing to the complex environment *in vivo*, these enhancers displayed a similar ability on the transdermal of FP, which further confirmed the results of the *in vitro* penetration. In contrast, there was no significant change in *T*_max_ and MRT between the enhancer containing group and the control group (*p* > .05). The *V* and Cl values of the enhancer containing groups were significantly reduced compared with the control groups (*p* < .05), which is conducive to maintaining the prototype component of the drug *in vivo*, and prolonging the action time.

**Table 2.** Pharmacokinetic parameters of FP enantiomers in rabbits after application of transdermal patches loaded *SR*-FP with or without enhancers.

parameters	Control		*d*-4-TER		*d*-4-T-dC18		*l*-4-T- dC18	
	*R*-	*S*-	*R*-	*S*-	*R*-	*S*-	*R*-	*S*-
*T*_max_ (h)	20.40 ± 3.92	21.20 ± 4.13	21.67 ± 7.86	18.00 ± 8.00	26.00 ± 0.00	24.00 ± 2.00	19.33 ± 5.70	25.33 ± 0.67
*C*_max_ (μg/mL)	0.89 ± 0.05	1.22 ± 0.09	1.67 ± 0.10*	3.06 ± 0.24*	2.72 ± 0.35*	4.74 ± 0.96*	4.43 ± 1.03*	8.27 ± 2.49*
AUC_0-_*_t_* (h·μg/mL)	17.71 ± 1.05	26.41 ± 1.66	46.07 ± 4.55*	92.10 ± 10.58*	72.28 ± 9.93*	129.15 ± 28.98*	110.62 ± 21.62*	209.04 ± 54.76*
*V* (L)	342.28 ± 15.81	414.21 ± 25.00	251.59 ± 56.91	99.27 ± 18.81	139.60 ± 34.29	92.94 ± 28.69	65.31 ± 28.90	68.51 ± 17.97
Cl (L/h)	23.46 ± 1.72	12.69 ± 2.00	5.72 ± 0.58	3.79 ± 0.72	4.63 ± 0.14	2.61 ± 0.46	4.06 ± 0.77	2.43 ± 0.75
MRT (h)	17.93 ± 1.26	21.52 ± 0.46	22.52 ± 0.64	22.17 ± 0.18	23.92 ± 1.53	23.92 ± 1.10	20.43 ± 0.29	20.91 ± 0.54
*F* (%)	3.86	2.84	10.05	14.87	15.77	20.86	24.13	33.76
*E_f_*	1.00	1.00	2.60*	3.49*	4.08*	4.89*	6.25*	7.92*

Data are given as mean ± SE (*n* = 6).

Absolute bioavailability (*F*) calculated as follows: AUC_patch_·Dose_i.v._/AUC_i.v_·Dose_patch_.

Enhancement factor (*E_f_*) calculated as follows: AUC_0-_*_t_* (with enhancer)/AUC_0-_*_t_* (without enhancer).

*Significantly different from FP in control (*p* < .05).

Absolute bioavailability (*F*) was calculated by the following equation:

$$F={\mathrm{AUC}}_{\mathrm{patch}}\cdot {\mathrm{Dose}}_{i.v.}/{\mathrm{AUC}}_{i.v.}\cdot {\mathrm{Dose}}_{\mathrm{patch}}$$

Usually, transdermal patches could obtain the *F* of more than 40%, however, the *F* values of developed patch in our study were less than 35%, it may attribute to the quantities of drug left in the peeled patch. As the content of the drug in the patch was detected after removing at 24 h, more than 50% of drugs had been detected.

#### Analysis of *in vitro* and *in vivo* correlation

IVIVC was defined as forecasting models characterizing the relationship between *in vitro* penetration and *in vivo* plasma concentration for a given drug. For this purpose, the data of intravenous administration and patch administration were deconvoluted to calculate the drug concentrations in plasma, and the *vivo*% and *vitro*% were then calculated according to the experimental results. Some of the enhancers were selected as representatives for IVIVC prediction, the establishment of point-to-point correlation function was built between the *in vitro* permeation data and *in vivo* absorption for FP enantiomers.

The correlation coefficient indicated excellent correlation, the exceptional IVIVC relationship indicated that the results of *in vitro* experiment could be used in the further study of patch system, presented in supporting information Figure S2. The correlation analysis using the GraphPad Prism 7.04 program evinced that the penetration amount of the drug from patches *in vivo* demonstrated a relatively good correlation with the cumulative amount of FP from patches *in vitro* when using enhancers (*r* > 0.8783), which indicated that *in vitro* isolated study was reliable to predict FP *in vivo* dynamics absorption.

#### *In vitro* drug release experiment

*In vitro* release experiment was carried out to explore the role of chemical enhancers in *SR*-FP release from patches. As is presented in supporting information Figure S3, due to the small error in the *in vitro* release experiment, the error line in the figure is not obvious. The cumulative percentages of *SR*-FP release from patches within 24 h for the control group, *d*-4-TER group and *l*-4-TER group were 70.15 ± 0.75%, 66.58 ± 1.57% and 69.63 ± 1.06%, respectively. There was no significant difference in the release amount among the above three groups (*p* > .05), however, the cumulative release percentage of *l*-4-T-C7 and *l*-4-T-dC18 were 78.29 ± 0.8% and 91.09 ± 1.65%, respectively, which were significantly higher than that of *l*-4-T-C7 and *l*-4-TER. It was noteworthy to point out that the effects of 4*-O-*acylterpineol on *SR*-FP release were consistent with the *in vitro* transdermal experiment and *in vivo* pharmacokinetic study. Usually, it is regarded that the transdermal delivery of drug in the patch includes two processes: release and penetration. The present result suggests that the promoting effect of enhancers depends not only on the promoting ability through the skin but also on the release speed from the patch.

In general, drug-PSA interaction and the molecular mobility of PSA were considered as two main factors that influenced the drug release process (Morimoto et al., 1992), drug-PSA interaction generally plays a more important role in the drug release (Liu et al., 2016; Song et al., 2016). It could be deduced in the present study that 4-*O*-acylterpineol promoted the release process of FP mainly by increasing the molecular mobility of PSA rather than decreasing the interaction between FP and matrix. The results of *in vitro* release indicated that hydrogen bond between FP and PSA was not formed due to neither *l*-4-TER nor *d*-4-TER could improve the release percentage of FP compared to the control group. Therefore, drug release from patches might mainly be dominated by PSA molecular mobility. So, it is postulated that higher molecular mobility of PSA would introduce a higher formation frequency of free volume in the PSA, thereby enhancing the FP release from PSA according to the free volume theory (Song et al. 2016). Certainly, further investigation such as thermal analysis and rheological tests should be conducted in future to prove this hypothesis.

### Molecular simulation

The details of molecular interaction between enhancers and the CERs which are confirmed as the major constituents forming the skin barrier in the lipids of SC were explored by molecular simulation. Molecular simulation is an effective tool to characterize the interaction between lipid barrier and drugs, as well as to show dynamic properties of enhancers. (Liu et al., 2017b). As a kind of ceramide, CER-NP could be synthesized by nonhydroxy acid (N) and phytosphingosine (P). Since CER-NP exists in the cuticle of various biological skin, it was often used to inspect the influence of enhancers on the lipid barrier. The minimum energy complex of the system was computed using Materials Studio software and the results were presented in Figure 3.

As in Figure 3(A,B), the hydrogen bond was discovered between the carbonyl group of FP enantiomers and the polar head region of CER-NP, but the docking energy of *R*-FP and *S*-FP were only –0.998 and –0.935 kcal/mol. In the enhancer-drug-CER ternary system, the hydrogen bond was observed between enhancers and CER-NP in Figure 3(C–N). The calculated hydrogen-bond energy of all the systems was shown in supporting information Table S1, the bond energy was generally negative, indicating that the interaction of CER-NP with the FP or enhancers was possible. In the chiral 4-*O*-acylterpineol-FP enantiomer-NP system, the hydrogen bond was observed between chiral 4-*O*-acylterpineol and CER-NP instead of between FP enantiomer and CER-NP. It means that enhancers had a better affinity to the polar head of the CER-NP than FP enantiomers because of the higher absolute values in the docked energy. It could offer a reasonable explanation for promoting the penetration of drugs that enhancers increased the skin permeation of FP enantiomers mainly by increasing the mobility of the SC intercellular lipids and decreasing the interaction between FP enantiomers and the SC intercellular lipids (Li et al., 2017; Liu et al., 2019). It was also found that the hydrogen bond energy for *d*-4-TER of the ternary system was higher than that for *l*-4-TER of the ternary system, the results were also confirmed by the *in vitro* and *in vivo* experiment.

As far as the other enhancers (*d*-T-dC18, *l*-T-dC18, *d*-T-C14, *l*-T-dC14) were concerned, which formed hydrogen bond interactions with the polar head of the CER-NP assemblies, the experimentally determined ER increased in line with absolute values of docked energy. As the amount of FP which can penetrate through the skin increases, the enhancer binds more tightly with the CER, and the energy of hydrogen bonding increased to establish a positive correlation between ER and docked energy. It can be deduced that enhancers are combination with SC by hydrogen bond, the higher the docked energy between compounds and SC, the stronger promoting abilities that can be obtained in patch for permeant dissociate from the polar domain of CERs. To prove this hypothesis requires further investigation.

#### ATR-FTIR study

**Figure 4.**
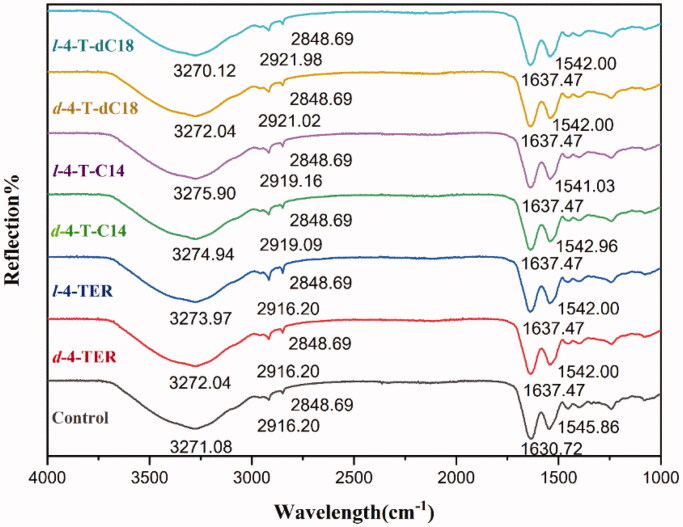
ATR-FITR spectra of the rabbit SC after treatment with or without enhancers (*n* = 3). The OH symmetric vibration of SC lipids is around 3271 cm^–1^, the initial frequencies of *v_s_*CH2 and *v_as_*CH2 are around 2848 cm^–1^ and 2916 cm^–1^, respectively. The amide I and amide II are around 1640 cm^–1^ and 1543 cm^–1^, respectively.

To further determine the interaction sites between enhancer and SC, ATR-FTIR was selected to provide changes of the surface layer of the skin (Figure 4). 4-*O*-acylterpineol including *d*-4-TER, *l*-4-TER, *d*-4-T-C14, *l*-4-T-C14, *d*-4-T-dC18 and *l*-4-T-dC18 were selected as representative enhancers for ATR-FTIR investigation. As far as the control group was concerned, the OH symmetric vibration of SC lipids is around 3271 cm^−1^, the initial frequencies of *v_s_*CH_2_ and *v_as_*CH_2_ are around 2848 cm^−1^ and 2916 cm^−1^, respectively. The SC keratin showed two sharp amide vibrations, amide I (C = O stretching of the peptide bond, around 1640 cm^−1^) and amide II (mainly N-H bending mode of vibration, around 1543 cm^−1^) (Tatulian, 2019).

**Figure 5.**
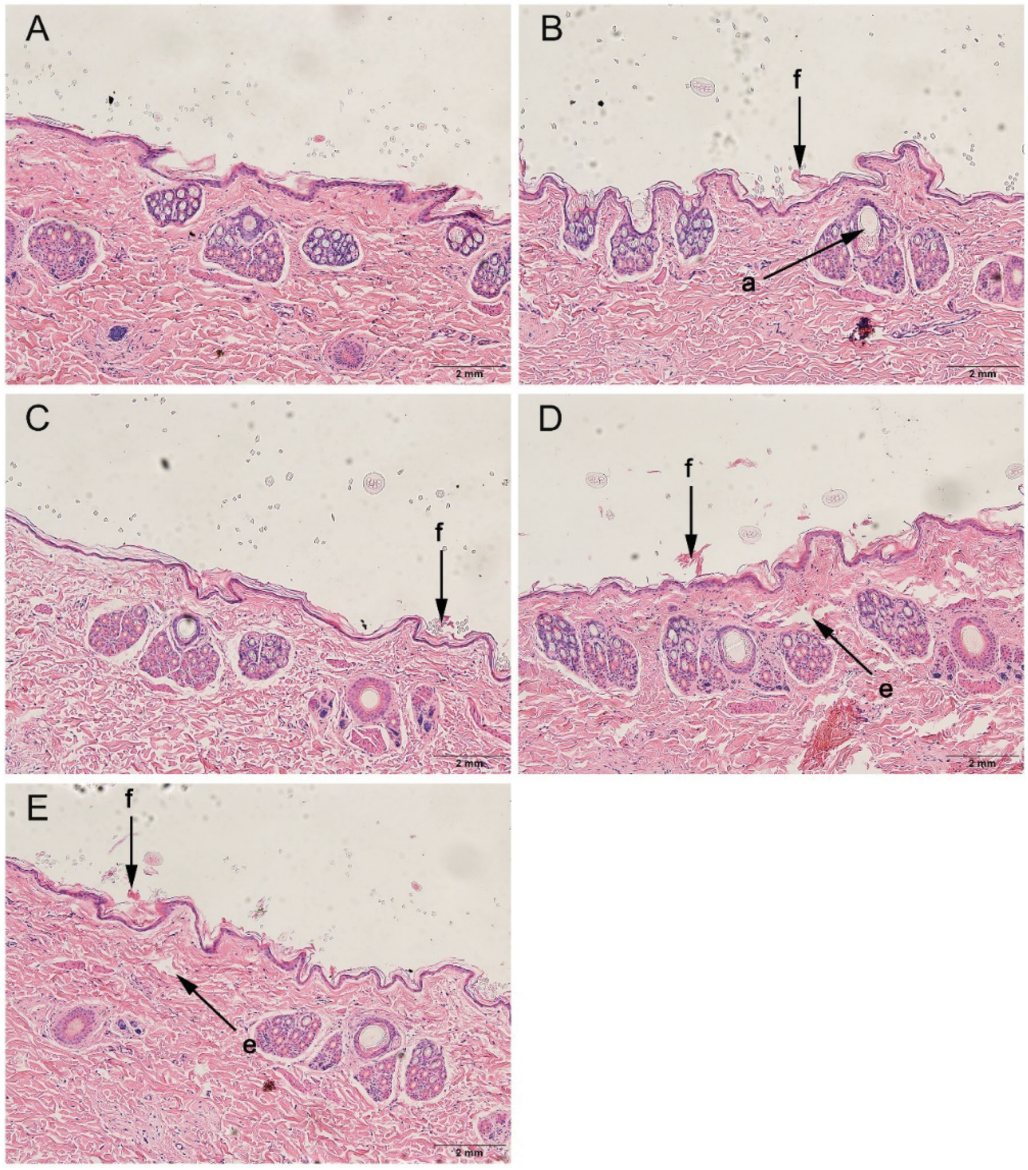
Histological sections of rabbit skin after treatment with or without enhancers. (A): control, (B): *d*-4-TER, (C): *l*-4-TER, (D): *d*-4-T-dC18 and (E): *l*-4-T-dC18. All tissue sections are magnified 100× (H&E). a, appendageal dilatation; e, edema; and f, focal disruption of epidermis, hyperkeratosis.

The results indicated that nearly all chiral 4-*O*-acylterpineol underwent a higher shift in the stretching vibration peak position of OH. However, the peak of *l*-4-T-dC18 shifted to a lower wavenumber. The rabbit skin treated with chiral 4-*O*-acylterpineol produced a higher shift in *v_as_*CH_2_ vibration peak positions, compared with the control group, however, neither *d*-4-TER nor *l*-4-TER experienced any shift in the peak of *v_as_*CH_2_. Meanwhile, chiral 4-*O*-acylterpineol made a higher shift in the stretching vibration peak position of amide I and a lower shift in the stretching vibration peak position of amide II compared to the control group.

The frequency of OH symmetric stretching vibration is between 3200-3300 cm^−1^ (Moore and Rerek, 2000). The shift to looser arrangement occurred in the skin lipid when OH vibration peak shifted to a higher wavenumber, implying the increased hydration of the SC (Csizmazia et al., 2012). It is generally regarded that the increasing permeability of drugs due to the higher shift of OH stretching vibration in SC created by enhancers (Okeke and Boateng, 2016). The results exhibited that when the rabbit skin had been treated with *l*-4-T-C14, the OH vibration increased from 3271.08 cm^−1^ to 3275.90 cm^−1^, implying the increased hydration of the skin lipid. It could be found that the ability of 4-T-C14 to permeate the drug was higher than that of 4-TER and the control group. However, the 4-T-dC18 which had higher promoting ability than 4-T-C14 caused lower spectral shift of OH stretching vibration than that of the 4-T-C14, even the *l*-4-T-dC18 which had the highest penetration effect caused lower spectral shift of OH stretching vibration than that of control. It was indicated that another mechanism plays an important role in penetration of 4-T-dC18, which still needs exploring in depth.

It was reported that terpenes introducing lipid alkyl chains exert their enhancing effect both upon disrupting the hydrogen bond network of the lipid bilayer and increasing the mobility of the SC intercellular lipids (Can et al., 2013; Liu et al., 2017a). The ATR-FTIR results showed the wavenumbers of *v_s_*CH_2_ and *v_as_*CH_2_ had no changes in the presence of 4-TER as enhancers, however, the wavenumber of *v_as_*CH_2_ to higher frequency occurred in the presence of 4-*O*-acylterpineol as enhancers. This result suggested that the permeation effect of 4-*O*-acylterpineol could involve the disrupted SC lipids due to its lipid alkyl chains insertion into the lipid domain of stratum corneum. Meanwhile, it could be concluded that the ability of 4-T-dC18 to disorder the SC lipids was more potent than that of 4-T-C14, and the wavenumber of *v_as_*CH_2_ showed the highest drug flux for *l*-4-T-dC18, which was in agreement with our *in vitro* skin permeation data. It may be due to the relatively long lipid alkyl chain and the double bonds of increase lipophilicity, which caused the increase of lipid mobility and the decrease of the diffusional resistance of permeant.

The bands of amide I and amide II presented information about the SC protein. It was observed that amide I bands can be attributed to the β-sheet structure (1637–1613 cm^−1^) which was formed by the uncoiling of the α-helical structure (1662–1645 cm^−1^) for various systems (Sarroukh et al., 2013), displaying that the secondary structure of proteins in the skin changed. Comparing 4-*O*-acylterpineol with the control group, the amide I bands demonstrated significant variation from about 7 cm^−1^ to higher wavenumbers and amide II shifted from about 3 cm^−1^ to lower wavenumbers after application of 4-*O*-acylterpineol. Therefore, 4-*O*-acylterpineol may transform the α-helical structure to β-sheet structure, thereby making the corneocytes more permeable.

As a whole, the wave number shifts outlined above indicate that increased transdermal flux of FP by enhancers correlate with increased SC lipid mobility, the hydration of OH, and the SC protein interaction caused by exposure to 4-*O*-acylterpineol. The higher promoting activities of 4-T-dC18 mainly correlates with SC lipid fluidization. The difference between *d*-4-*O*-acylterpineol and *l*-4-*O*-acylterpineol cannot be clearly observed through the ATR-FTIR, however, it can be achieved by molecular simulation techniques.

### Histological observation

The histological evaluation was employed to gain insights into the physicochemical changes of the skin and roughly evaluate the skin irritation of enhancers. Histological studies of the optimized enhancer group (chiral 4-T-dC18) and normal enhancer group (chiral 4-TER) were performed and compared with the control group (supporting information Figure 5).

The control group exhibited an intact morphology throughout the skin of epidermal keratinocytes. When chiral 4-*O*-acylterpineol were used as enhancers, there was marginally focal disruption of the epidermis with thinning, which might be related to the conformational changes of the lipid bilayer. Meanwhile, skin injury also observed with chiral 4-T-dC18 showed slight dermal edema. Although the chiral 4-*O*-acylterpineol induced a mild impairment of the barrier function of the skin, it did not alter the viability of the skin, meanwhile, the impairment was reversible. The reduced skin barrier is reflected in the increased permeability of the skin and therefore increased flux (Prasad et al., 2007, 2009). Results of the histological evaluation further support higher permeation with chiral 4-*O*-acylterpineol. Meanwhile, the FP patch containing chiral 4-*O*-acylterpineol was safe for application on the skin, and the marginally pathological changes have no effect on skin integrity, which was in line with the expectation of chiral 4-T-dC18 derived from the terpene natural compound.

## Conclusion

From the results of present investigations, *l*-4-T-dC18 was shown to be the most promising enhancer for increasing the transdermal amount of *SR*-FP and its enantiomers, with the superiorities of high flux and low skin irritation. The IVIVC analysis revealed that there was a good correlation for permeants between the penetration amounts from patches *in vitro* and the AUC_0-∞_ *in vivo* when employing 4-*O*-acylterpineol derivatives as penetration enhancers. Moreover, the mechanistic insights studies suggested that chiral 4-*O*-acylterpineol could make lipids and keratin looser or more permeable, and bind more tightly with the CER-NP to decrease the affinity between FP enantiomers and the lipids in the SC. In addition, this investigation proposes a new idea for designing chiral penetration enhancers, and provides a reference for improving the transdermal delivery of chiral drugs through rationally selection of chemical enhancers.

## Supplementary Material

Supplemental Material
