# Supplementary material for: Chiral 4-O-acylterpineol as transdermal permeation enhancers: insights of the enhancement mechanisms of a transdermal enantioselective delivery system for flurbiprofen
Source: Drug Deliv. 2020 May 13;27(1):723–35. doi: 10.1080/10717544.2020.1760403 (PMC7269032; doi:10.1080/10717544.2020.1760403)
Supplement: Supplemental Material [file IDRD_A_1760403_SM3859.docx]

**Figure S1.** The structure and synthetic route of chiral 4-*O*-acylterpineol (A) *d*-4-terpineol (B) *l*-4-terpineol (C) *d*-4-*O*-acylterpineol (D) *l*-4-*O*-acylterpineol.

**Figure S2.** *In vitro* and *in vivo* correlation analysis of *SR*-FP or its enantiomer in patches with or without enhancers: (A)~(B) Control; (C)~(D) *d*-4-TER as promoter; (E)~(F) *d*-4-T-dC18 as promoter; (G)~(H) *l*-4-T-dC18 as promoter. (*S*-FP represented by the blue circle; *R*-FP represented by the red circle).

**Figure S3.** Cumulative release proﬁles of the *SR*-FP from the patches with and without the permeation enhancer (*n* = 4, Mean ± SD).

**Table S1** The calculated hydrogen-bond energy of each group

**Figure S1.**


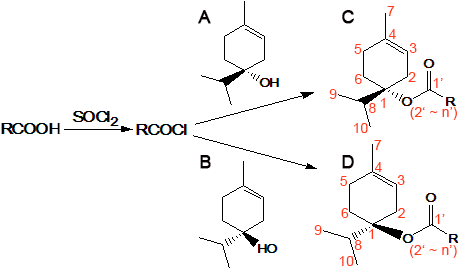


**Figure S2.**


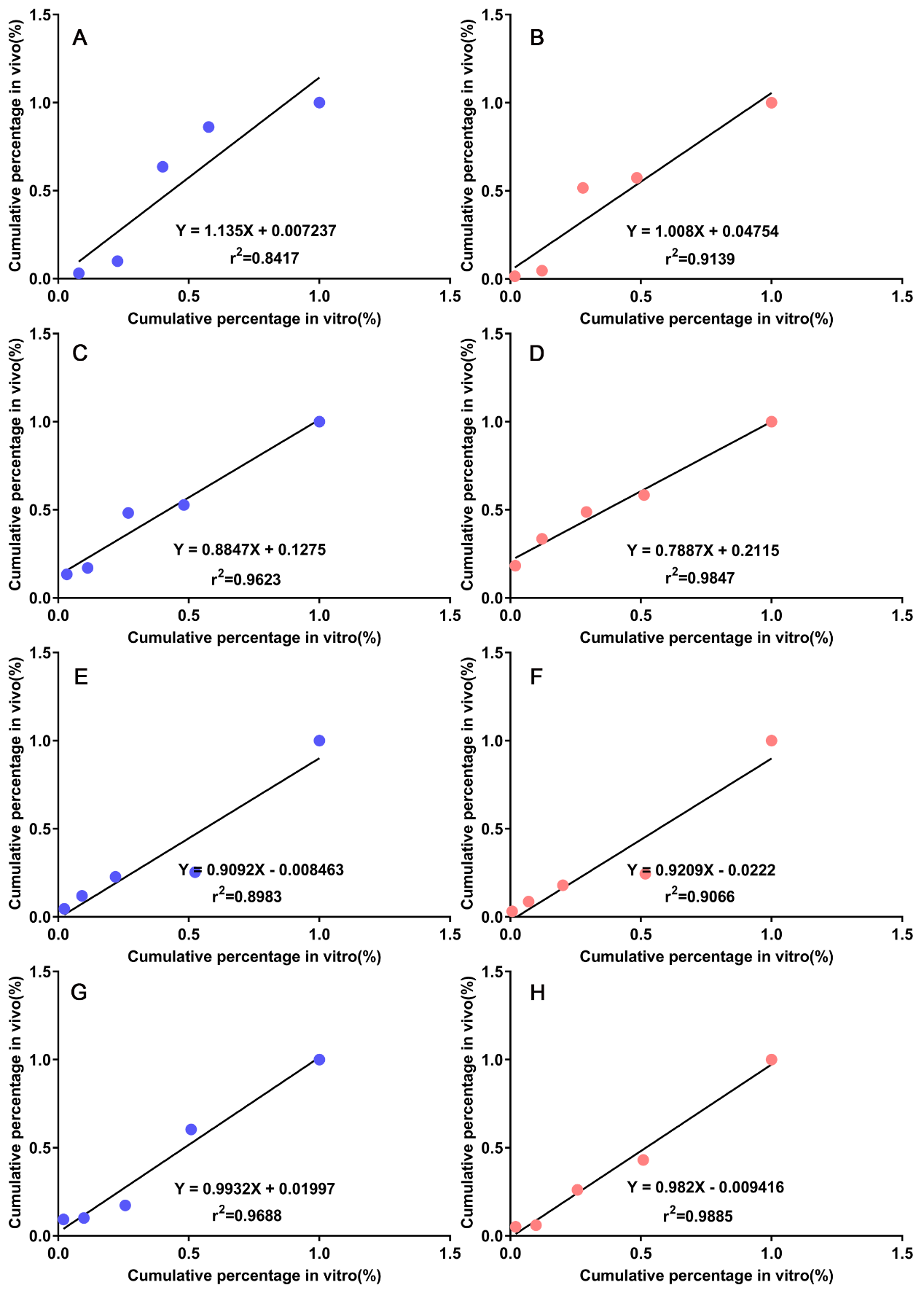


**Figure S3.**


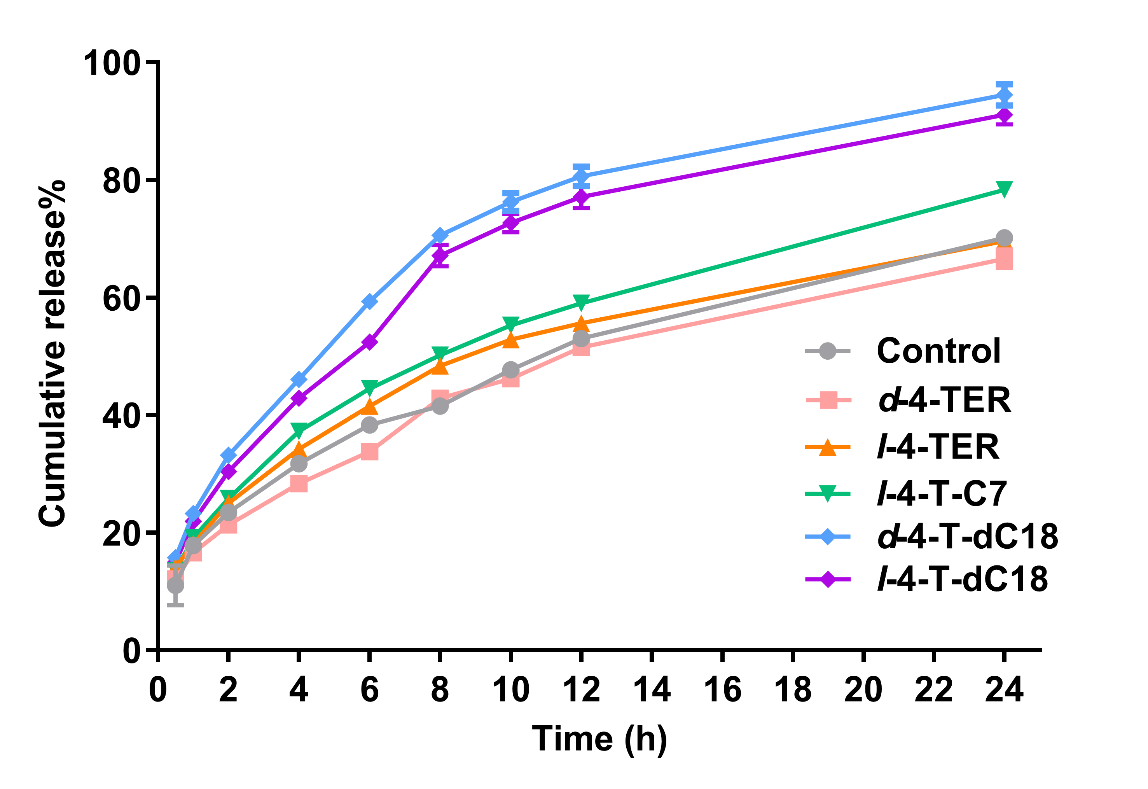


**Table S1** The calculated hydrogen-bond energy of each group

| Group | Hydrogen-bond energy | Group | Hydrogen-bond energy |
| --- | --- | --- | --- |
| *R*-FP-NP | -0.998 | *S*-FP-NP | -0.935 |
| *R*-FP-*d*-4-TER-NP | -1.273* | *S*-FP-*d*-4-TER-NP | -1.318* |
| *R*-FP-*l*-4-TER-NP | -1.130* | *S*-FP-*l*-4-TER-NP | -1.183* |
| *R*-FP-*d*-4-T-C14-NP | -1.464* | *S*-FP-*d*-4-T-C14-NP | -1.363* |
| *R*-FP-*l*-4-T-C14-NP | -1.334* | *S*-FP-*l*-4-T-C14-NP | -1.273* |
| *R*-FP-*d*-4-T-dC18-NP | -1.573* | *S*-FP-*d*-4-T-dC18-NP | -1.628* |
| *R*-FP-*l*-4- T-dC18-NP | -1.862* | *S*-FP-*l*-4- T-dC18-NP | -1.872* |

The unit of hydrogen-bond energy is kcal/mol

“*” is significantly different from binary system for corresponding drugs, *p* < 0.05
